# Supplementary material for: Modeling diffusive search by non-adaptive sperm: Empirical and computational insights
Source: PLoS Comput Biol. 2025 Apr 17;21(4):e1012865. doi: 10.1371/journal.pcbi.1012865 (PMC12005489; doi:10.1371/journal.pcbi.1012865)
Supplement: Attachment — Submitted filename: Response_to_Reviewers_auresp_2.docx [file pcbi.1012865.s004.docx]

Editor’s Comments

1) We have noticed that you have uploaded Supporting Information files, but you have not included a complete list of legends. Please add a full list of legends for your Supporting Information files after the references list.

Response: The response to Reviewer #2’s comments resulted in moving information from the supplement back into the main results of the manuscript. In this revised version, there are no supplementary materials to include.

2) Please amend your detailed Financial Disclosure statement. This is published with the article. It must therefore be completed in full sentences and contain the exact wording you wish to be published.

- State the initials, alongside each funding source, of each author to receive each grant. For example: "This work was supported by the National Institutes of Health (####### to AM; ###### to CJ) and the National Science Foundation (###### to AM)."

- State what role the funders took in the study. If the funders had no role in your study, please state: "The funders had no role in study design, data collection and analysis, decision to publish, or preparation of the manuscript.".

If you did not receive any funding for this study, please simply state: u201cThe authors received no specific funding for this work.u201d

Response: We have amended the financial disclosure statement.

Reviewers' comments:

Reviewer #1: The paper presents computational results that support empirical methods for assessing sperm fitness within assisted reproductive technologies. The authors conceptualize the physiological function of sperm as a diffusive search process and develop computational tools to explore the underlying causal dynamics of sperm fitness. They introduce both a probabilistic measure of sperm fitness and an information-theoretic measure of sperm selection, each situated within a relevant theoretical framework. The figures, methods, and explanations collectively support the idea that the developed agent-based models present compelling arguments, offering valuable insights toward a theory of sperm selection. Overall, the references and detailed results are consistent and well-explained. The responses to reviewer comments have strengthened the paper's main mathematical results and established stronger connections with relevant empirical work.

The comments raised by reviewers were adequately addressed, and the main issues have been resolved. Below are additional comments and questions regarding the responses to the original reviewer feedback:

Comments on the GitHub Repository Updates

From Document: "Response: We have updated the movement function description in the paper to make the model implementation more clear as well as to address potential issues with compounding random noise in the initial models. The movement functions are described in the materials and methods. We have modified Figure 1 and no longer include the simulations with simple Gaussian walks in lieu of simulations with the updated movement functions. We have updated the code in the GitHub repository accordingly."

Comment: Although the responses indicate that the code source at https://github.com/CAS-ReproLab/Fertilization_ABM has been updated, the GitHub repository itself has not received any updates in the past six months. It is possible that updates have been made to a nonpublic version of the code but have not yet been pushed to the public repository. Please clarify if there is a new link or specify which codes have been updated.

Response: Thank you for pointing this out. We sincerely apologize. We did update the code, and as the reviewer guessed, we mistakenly did not commit the changes to the Github repository prior to the review. The code base has been updated with the current version for all relevant models.

From Document: "Response: Not sure I fully understand this critique, so apologies in advance if the response is off base. In sperm analysis, summary statistics are often used to describe sperm phenotypes (e.g., motility measures, etc.) without paying much attention to the underlying distributions, which in our experience are often not symmetric. We feel that this perspective on sperm physiology misses the point that the underlying distributions matter greatly, and ignoring this likely contributes to diagnostic shortcomings and misconceptions about the physiological tasks that sperm perform. The approach to quantifying fitness and the magnitude of selection may be an important step toward improving sperm diagnostics and understanding the constraints that influence which sperm will ultimately fertilize an egg. We realize that the idea is mostly descriptive and preliminary, but we are working on a 'more detailed analysis of the underlying statistical considerations as well as the information-theoretic implications of selection during fertilization.'”

Comment: The more detailed analysis that is being worked on, I am assuming that is referring to a future work. For this comment it would be useful to address the current information theoretic sections of the paper to make clear the distinction between what is present in the paper and the future proposed work. This distinction would both enhance the reader’s understanding of the scope and implications of the current research and ensure the reviewers are adequately understanding the points that you are getting across.

Response: Thank you for clarifying. To address the current information theoretic sections of the paper and make clear the distinction between what is in this paper and what will be in proposed work, we have included an updated discussion section with detailed discussion regarding how these measures may fit into the current demands of sperm fertility diagnosis and selection for assisted reproduction- (Lines 446-486). To allude to future work, we have added forecasting statements such as: ‘Additionally, it [relative information gain] lays the groundwork for a new biological context for reproduction by reframing the process of fertilization in information theoretic terms as a form of learning process[57]’.

Reviewer #2: While the authors did a good job in removing ambiguous passages and overstatements the presentation has now suffered in two ways. First when reading the results, it is not clear what the setup and setting is, what are the mazes ABC where the egg is, what the sperm is doing while moving in the model. I really do not understand why this information is now hidden in S1 figure (which still doesn’t contain mazes). Second negative effect is that by removing the overstatements there is now no any kind of qualitative explanation of the results (at least in the way I was trying to make sense of those results in my previous comment). So now we are only presented with some results (dependencies) but no attempt to rationalize them.

Taken together, while the over interpretations were removed, the clarity of results has suffered considerably. I do think this needs to be fixed before it can be published.

Response: Thanks for pointing this out, and apologies for the confusion. We tried to make a clearer distinction between the model setup in the methods and the model interpretation in the results section by separating the design considerations from the results. This clearly did not have the intended effect. So, we have moved the information from the supplement back into the main manuscript and reorganized the results section accordingly. We hope these changes will reinforce the logical flow of the manuscript and enhance the clarity of the results.
