# Supplementary material for: Modeling diffusive search by non-adaptive sperm: Empirical and computational insights
Source: PLoS Comput Biol. 2025 Apr 17;21(4):e1012865. doi: 10.1371/journal.pcbi.1012865 (PMC12005489; doi:10.1371/journal.pcbi.1012865)
Supplement: Attachment — Submitted filename: ReviewF.docx [file pcbi.1012865.s001.docx]

The paper presents computational results that support empirical methods for assessing sperm fitness within assisted reproductive technologies. The authors conceptualize the physiological function of sperm as a diffusive search process and develop computational tools to explore the underlying causal dynamics of sperm fitness. They introduce both a probabilistic measure of sperm fitness and an information-theoretic measure of sperm selection, each situated within the relevant framework.

The figures, methods, and explanations collectively support the idea that the developed agent-based models present compelling arguments, offering valuable insights toward a theory of sperm selection. Overall, the references and detailed results are consistent and well-explained.

Major Comments/Issues

-None

Minor Comments/ Issues

1- The codes used to generate the results in Fertilization_ABM are generally well-labeled and aligned with the figures in the document. However, some of the supporting code could benefit from additional comments, such as lines 22 and 134/135 regarding set size and mu. It’s unclear whether the mu on line 134 is the same as in Figure 1C, where it has a value of 0.3 in the included example code but 0.5 in the window panels.

i. A possible inclusion would be a text file for the parameters used for each of the frames in plot 1.

2- For Figure 1, a round particle of known dimension is mentioned. I have a few questions regarding this particle dimension:

i. Does the particle dimension affect the possible angular distribution?

ii. Were any investigations conducted into the particle's dimension relative to the maze proportions?

iii. Where is this parameter located within the generating code?

3 - In line 319, ANOVA is mentioned, while in line 611, it is fully introduced as "analysis of variance." Introducing it as "analysis of variance" in line 319 may be more fitting for consistency with the full introduction.

I. Since ANOVA is well-established, mentioning the full name may be unnecessary in either instance.
