# Supplementary material for: Modeling diffusive search by non-adaptive sperm: Empirical and computational insights
Source: PLoS Comput Biol. 2025 Apr 17;21(4):e1012865. doi: 10.1371/journal.pcbi.1012865 (PMC12005489; doi:10.1371/journal.pcbi.1012865)
Supplement: Attachment — Submitted filename: Response to Reviewers.docx [file pcbi.1012865.s003.docx]

We thank the editors and reviewers for their continued consideration and feedback. We have addressed each reviewers comments in-kind below. Some major revisions to the manuscript include, but are not limited to:

- Provided additional mathematical detail for the models.
- Modified figures and text to better separate the results section from the methods and materials section.
- Modified diagrams and figures to enhance clarity and rigor.
- Removed or modified statements in the text that were unclear or were flagged as over interpretations of the model.

**Reviewer #1:** The paper presents computational results that support empirical methods for assessing sperm fitness within assisted reproductive technologies. The authors conceptualize the physiological function of sperm as a diffusive search process and develop computational tools to explore the underlying causal dynamics of sperm fitness. They introduce both a probabilistic measure of sperm fitness and an information-theoretic measure of sperm selection, each situated within the relevant framework. The figures, methods, and explanations collectively support the idea that the developed agent-based models present compelling arguments, offering valuable insights toward a theory of sperm selection. Overall, the references and detailed results are consistent and well-explained.

Response: We thank you for your time, consideration, and thoughtful feedback. We have addressed the comments in kind below.

Major Comments/Issues

-None

Minor Comments/ Issues

1- The codes used to generate the results in Fertilization_ABM are generally well-labeled and aligned with the figures in the document. However, some of the supporting code could benefit from additional comments, such as lines 22 and 134/135 regarding set size and mu. It’s unclear whether the mu on line 134 is the same as in Figure 1C, where it has a value of 0.3 in the included example code but 0.5 in the window panels.

Response: WE have updated the movement function description in the paper to make the model implementation more clear as well as to address potential issues with compounding random noise in the initial models. The movement functions are described in the materials and methods. We have modified figure 1, and no longer include the simulations with simple Gaussian walks in lieu of simulations with the updated movement functions. We have updated the code in the Github repository accordingly.

i. A possible inclusion would be a text file for the parameters used for each of the frames in plot 1.

Response: The parameters for the sperm simulations are now described in S2 Table.

2- For Figure 1, a round particle of known dimension is mentioned. I have a few questions regarding this particle dimension:

i. Does the particle dimension affect the possible angular distribution?

Response: The particle dimension does not affect the angular distribution, but could affect the radial velocity due to the way that agent size is calculated in the native Netlogo functions. To ensure accuracy, we defined instructions in the portion of the program where the length scale is defined which described how to change the agent size if the length scale changes.

ii. Were any investigations conducted into the particle's dimension relative to the maze proportions?

Response: We only simulated at one length scale, where the dimensions of each grid square are 40x40 um. The maze proportions would undoubtedly influence the outcomes because the sperm agents movement is dependent on the time scale with accumulating Gaussian noise. We didn’t explore this explicitly, but intend to pursue scaling properties in similar models and empirical measurements in future studies.

iii. Where is this parameter located within the generating code?

Response: The particle dimensions are determined in the setup routine. The maze proportions are not automatically generated, because the mazes were constructed manually.

3 - In line 319, ANOVA is mentioned, while in line 611, it is fully introduced as "analysis of variance." Introducing it as "analysis of variance" in line 319 may be more fitting for consistency with the full introduction.

Response: We have updated the text to reflect analysis of variance (ANOVA) at first use, and ANOVA thereafter. Thanks for point this out.

I. Since ANOVA is well-established, mentioning the full name may be unnecessary in either instance.

Response: We agree, but felt if was important to define it at least once, just in case.

**Reviewer #2:** In this manuscript, the authors suggest to use simple modelling approach to study the selective pressure and variability in the sperm fitness during the fertilisation process. I actually do like the general idea and fully support the usage of simple enough models to make the point. Indeed with the help of tractable models it is possible to bring across very powerful mathematical predictions that can be the key to understanding even such complex phenomena as discussed in this manuscript. So while in general strongly supporting the simple idea, I have multiple technical comments/problems to the implementation which prevent me from being fully positive about this work. Below I provide the comments in the chronological order. I note the most critical points with a star * sign. Also there is a tendency to over-interpret the results from the models.

Response: We thank you for your time, consideration, and thoughtful comments. We fully agree that the initial report did not adequately reflect clear definitions of the model, and we feel that it has benefited substantially from incorporating mathematical clarity and literature background in response to your helpful feedback. Overall, we have made extensive changes to the original report and added more mathematical mathematical clarity to the model description, modified text in places were the meaning was determined to be ambiguous or trivial, added additional literature review and discussion about asymptotic behaviors of random walks, and rearranged the organization of the report so that the figures and details related to the model design and implementation are in the supplementary material which we feel makes it easier to separate model details from results. We hope that the following responses to your comments strengthen confidence in the report’s findings.

1. “Diffusive search is an intrinsic property of agents performing a random walk” - this is a strange statement, not all random walking agents are searching for something.

Response: We have removed this statement and replaced it with: “*Sperm-Agent Search is a Function of Ensemble Motility Pattern*” Line 82.

2. Fig 1 A is confusing, not clear what the blue, red and white parts are, also what is the zoom in square means. Maybe showing a photo of a real chip would help better than this schematics

Response: Thanks for pointing this out. Figure 1 has been thoroughly redesigned and moved to the supplementary material. We have updated the diagram of the model environment, the diagram of the agent movement functions, and replaced the simple Gaussian random walk examples with examples of the range of simulation behaviors the model can produce given combinations of parameters. Please see S1 Figure panels A-D.

3. Angle \theta in Fig. 1B is just showing the 90 degrees and not linked to the trajectory

Response: We have redrawn the diagram in S1 Figure B to highlight the movement function parameters and the addition of zero average Gaussian noise to each parameter.

4. "increasing the mean \mu .." - mean of what?

Response: This was initially the mean of the Gaussian distribution from which the noise was drawn. We have clarified the model description, and no longer refer to the mean in this way in the revised manuscript.

5. -"Minor adjustments to the mean or standard deviation of the distribution can have significant effects on the agents' diffusive search behavior" - this is very vague and unsubstantiated statement. What is minor, what is significant? Also search behaviour is not quantified in any way.

Response: We have removed this text from the report as well as clarified the model description and figure. S1 Figure A-D.

6. -"Agents with movement parameters that allow them to search space more quickly than others will exhibit a more rapidly increasing RMS” - this is also very vague statement. A walker moving along the straight line with constant speed will have higher asymptotic MSD - but will it search space more quickly? It is very imprecise formulation.

Response: We have removed this statement and updated our description of the model to enhance clarity.

7. -How random \delta is selected? can it be negative (if it comes from Gaussian distribution)?

Response: We apologize for the confusion. We have updated the description of the movement functions to clarify how the step sizes are generated. Lines: “413-429”

8. -“..search patterns to broader exploration of the surrounding environment ultimately resulting in altered diffusive search outcomes” - this is either unsubstantiated statement or trivial, should be removed in both cases.

Response: We have removed this statement in the revised report.

9. (*)-“Those with smaller ranges had fewer directions to go at each timestep and exhibited behavior that resembled crude progressive motility patterns (Figure 1E)” - how do we know? no reference, no figure

Response: We have removed the references to the simple Gaussian walks and moved the original figure 1 to supplementary material S1 Figure. We have removed this statement from the text.

10. -" Taken together, the results of these simplified models highlight the diffusive search functionality that emerges” - unsubstantiated - nothing definite has been said about search till this point.

Response: We agree that we overstepped with this statement. This text has been removed along with other references to the simple Gaussian walks. Instead we provide a more detailed description of the model and the effects of changing the movement function parameters. S1 Figure A-D

11. -"match the simulated Gaussian distributions from data” - not clear what it is, why to simulate Gaussian from data?

Only the means and standard deviations from the labeled CASA datasets were available, so we simulated the distributions using the available parameters to generate the distributions. We chose to use the data set that was available because it included labeled classification of the CASA parameters for categories of different motility types. This is described earlier in the paragraph on line 463 “Means and standard deviations of the movement parameters for each motility type were obtained from a previous report, and Gaussian distributions were simulated using Graphpad Prism.” We also made the following change to the sentence: Line 472 “Movement function parameters (VSL, VCL, and VAP) for the agents of each prescribed motility class were adjusted to approximate the simulated data”.

12. -Fig 2A: why experiment and simulations are shown at different magnification? It is impossible to see the tracks in simulations good enough to say if they are similar or not

Response: Thank you for point this out. We had originally included the 10X images so that multiple simulation trajectories could be seen at once. We didn’t do this for the live sperm because the motility types in the field of view are not uniform. However, we appreciate that this was confusing and have updated S2 Figure A to include temporally-coded images from the simulation that are at the same length scale as the microscopy time projection images.

13. (*)- "The resulting sperm motility quantitatively and qualitatively like mouse sperm..” how do we see that? based on which analysis? We see in Fig. 2 BCD that multiple quantifiers are off between data and simulations. Some have low values and we can’t tell how good/bad they are

Response: We have modified the description to be clear that the simulations do not exactly capture the movement properties of sperm. Line 475 “The distributions did not match exactly for every parameter or motility pattern, indicating that true sperm motility is more complicated that the simple agent rules in this report. Nevertheless, the resulting sperm motility patterns are qualitatively similar to mouse sperm and could be readily updated in future model iterations to accommodate alternative agent rules, or movement parameter distributions from other species if labeled CASA data is available.” On a side note: this was about the best we could do in this report due to the limitations of routine CASA data available to us. We have ongoing projects to address inadequacies in the motility measures, as well as measuring long trajectories in densely populated microscopy videos.

14. "These results demonstrate that the average diffusive search capability of a sperm population reflects the underlying distribution of sperm phenotypes. In other words, the ability to efficiently search space is an emergent property of the sperm population.” This is somehow very generic and trivial statement. The search efficiency was quantified with an arbitrary quantity (which is not really discussed who general it is) and then of course - different microscopic random walk leads to a different diffusion constant and to different exploration - of course? what else?

Response: We agree that this statement is trivial for readers who are familiar with random walks and/or active matter processes. However, we do want to be able to target a reproductive biologist audience as well with this report. This was part of our motivation for making the models mathematically minimal. The current understanding of sperm biology has largely focused on the competitive contribution of sperm characteristics to fertilization and organismal development (i.e., sperm competition and sexual selection). There has been a fair amount of research on how individual sperm cells move, but very little discourse on how statistical variation in sperm characteristics influence their fitness especially in the context of microenvironmental constraints on fertilization. We hope that is a sufficient reason to make a few otherwise trivial statements throughout the report, the statements genuinely may not be trivial to the typical experimental cell biologist. That said, we added a section in the discussion to address some of the well described asymptotic properties of correlated random walks, as well as to clarify the intention of this report. line 261 “The goal of this report is not necessarily to derive new findings on random walk behavior, but rather, to frame the physiological context of the sperm search for an egg using tools from this field with the hope of illuminating improved fertility analysis and/or prediction.”

15. The plots for the MSD, within the realm of the random walk model used can be fully analytically quantified, in particular by the MSD of the Ornstein-Uhlenbeck process that can be derived from the run-and-tumble-like random walk model.

Response: Thank you for pointing that out. This may be partially addressed by the removel of much of the original figure 1. That said, we avoided analytical derivations in this report in order to keep the models simple for a broad interdisciplinary audience, but we are currently working on analytical properties of sperm search in separate projects.

16. -"The simulations presented thus far predict that sperm populations will function on average to search all the space that they occupy, and the kinetics of the search process depend on the distribution of sperm motility patterns within the cell population” - There is nothing to predict! The result is known (trivial) analytically as soon as the model is formulated? Rephrase!

Response: We have removed this statement, and have attempted to clarify how known properties of random walks may inform sperm biology in the revised discussion section.

17. -Figure 5. I have difficulties with quantitative interpretation of these results, as the space/time scale of the experiment is unclear, and very difficult to tease out from the methods section. Also form that moment on some quantities start to be shown stochastically with units/dimensionless.

Response: Apologies for the confusion. We have revised the methods section to clarify the space and time scales of the models and clarified the units in subsequent figures. Please see ‘system boundaries’ lines “”394- 429’. Figure 2B has been revised to include plots of the area searched at first contact with the egg (um^2) rather than percentage of total searchable area. The time units are in seconds. Please note that this figure has been moved to Figure 1 in the revised manuscript as some of the earlier figures from the original submission have been moved to the supplementary material.

18. (*)-Results in Figure 5 in essence have clear, analytical explanation. For the open chamber the search time is the first passage time of the random walk to the target. And the plots show how that changes for a finite number of trajectories. The shortest possible time is the movement of the random walker on a straight path (very rare event). For an infinite number of realisations such a trajectory will always appear and thus provide a narrow cloud of points for 100 trials - this is an asymptotic value towards which the times converge. This behavior is to be expected as soon as the number of trajectories (particles) times the statistical probability of such trajectory to occur (can be calculated, like the authors did for labyrinths) will become equal to 1 - so one trajectory is enough. For smaller N of course we have to wait till whatever trajectory first reaches the target (thus a scatter and higher target hitting time).

Response: Thanks for pointing this out! We are currently pursuing the analytical properties of asymptotic sperm random walks as well as time averaged versus ensemble averaged behavior in separate project. As mentioned earlier, adequately addressing this requires more accurately fitted movement function parameters, and we are working on that now. That said, we have added additional reference to fundamental random walk properties in the discussion section.

19. Similarly - plots in B show the interplay of the time it takes to reach the target versus the number of distinct sites visited by the random walker. Reaching the target depends on the pdf of the particles to reaching out beyond a given distance with high enough total probability. In that there is a square root relationship of the distance and the time it takes to reach the target (if we argue in terms of time scaling). Another quantity is the number of distinct sites visited by the collection of random walkers. The scaling of this quantity for large N (number of walking particles) has been studied in 90s (see for example Larralde et al PRA 1992). The number of distinct sites visited with time (depending on the regime) scales at least linearly. So therefore it is also expected (from analytical arguments) that the number of sites visited with the increasing number of walkers will increase reaching to the 100% before the run is terminated when one of the particles hits the target.

Response: Thank you for pointing this out. We have now referenced this and a few other of Hernan Larralde’s foundational papers. N-sites and first passage times have featured heavily in our discussions for ongoing projects. One of the major challenges is that the sperm change motility over time and in ways that are heterogeneously distributed throughout cell populations. We are currently addressing this issue and plan a follow up report soon.

20. "Taken together, these results predict that diffusive search by non-adaptive sperm will exhibit a non-linear relationship with sperm density and that the role of chance in finding shortest path to the egg is modified by the spatial complexity of the microenvironment”. So, as before, I don’t think there is any need in the numerical simulations to predict the behaviours observed/shown in the plots. The qualitative outcome can be understood based on the known results from basic random walk properties. The only novelty (which is also probably difficult to straightforwardly derive analytically) are the results for labyrinth geometries. Those however, have no qualitative explanations, for example, with respect to the characteristic time scales, etc. So these are not predictions, but a simple numerical verification of the analytically predictable (interpretable) results.

Response: We understand this reservation, but also feel that there is some illustrative benefit for the empirically minded biologist readers in observing the results of the numerical simulations. Additionally, a later goal of this report is to highlight probabilistic measures of sperm fitness that can be tested empirically in follow up studies, so we hoped that the numerical simulations would serve here as ‘synthetic data’ to highlight the potential of those approaches. We also aim to convince biologist readers that the widespread conception of fertilization as a competition or race to and egg is built on some assumptions that are likely false, but will need a re-tooling of the kind of measurements we make when we study fertilization empirically.

21. "with a Markov transition matrix “ - this is not defined, also in the Methods it is hard to understand how exactly it looks like

Response: Apologies for this oversight. We have added the Markov transition matrix in S2 Table.

22. "Simulations consisted of 100 sperm, which was chosen as a reasonable (minimal) number” - based on what?

Response: Apologies for the confusion. We have updated the text to reflect how the number of simulations were chosen. Line 175 “Simulations consisted of 100 sperm, which was chosen as a reasonable (minimal) number that supported consistent random phenotype distributions across simulation runs, determined by increasing the size of sampled distributions until the observed mean approximately matched the ideal mean of the distribution from which the sample was drawn.”

23. -" with mean search time differences as large as ~627 seconds in the most extreme case” as mentioned above, the jumping between unit and unitless quantities - and no idea of the actual searching area - it is hard to give a physical meaning to the numbers provided.

Response: We have revised the plots in Figure 2 to reflect the area (um^2) at first contact with the egg rather than the % total searchable area.

24. (*) - "Taken together, the results from these simulations reinforce the conclusion that simple spatial hindrance by the latent structure of the microenvironment combined with variation in individual sperm phenotypes exerts quantifiable selective pressure on sperm. The fitness can be represented as an inferential probability of ‘success’, and the magnitude of selection can be represented using relative information gain.” I think this is the part which mixes the relatively straightforward predictions of the model (which are essentially what they are as soon as parameters are fixed) and the pressure/selection are the criteria imposed by the authors, and the more complex evolutionary concepts of the underlying biological problem. Instead, what authors should do - just explain their mathematical results, and then map them in the language of pressure and selection, but not mixing them (as also was done in the whole last section).

Response: Not sure I fully understand this critique, so apologies in advance if the response is off base. In sperm analysis, summary statistics are often used to describe sperm phenotypes (e.g., motility measures etc) without paying much attention to the underlying distributions, which in our experience are often not symmetric. We feel that this perspective on sperm physiology misses the point that the underlying distributions matter greatly, and ignoring this likely contributes to diagnostic shortcomings and misconceptions about the physiological tasks that sperm perform. The approach to quantifying fitness and the magnitude of selection may be an important step toward improving sperm diagnostics and understanding the constraints that influence which sperm will ultimately fertilize an egg. We realize that the idea is mostly descriptive and preliminary, but we are working on a more detailed analysis of the underlying statistical considerations as well as the information theoretic implications of selection during fertilization.

25. "Previous work modeling sperm search times in both 2D and 3D environments detailed several potential scaling laws for the diffusive search process relating search time to sperm number[38]” - the work cited, which is indeed based on a large body of analytical work, actually argue that the relevant movement of cells is not diffusive as considered in this work but along the straight paths which only reflect from the boundaries of the confinement. This needs to be commented.

Response: Apologies for this oversight. We have clarified that the reference study used rectilinear motion in their models in the revised text. Line 283 “Previous work modeling sperm search times in both 2D and 3D environments detailed several potential scaling laws for the diffusive search process relating search time to sperm number[36]. Similar to our observations in this report, a non-linear relationship between sperm number and search time was described, and the scaling laws depended on the dimensions of the search space. However, those simulations used sperm with constant velocity, rectilinear motion, and did not explicitly account for phenotypic heterogeneity or motility pattern changes over time.”

26. " The simulations in this report predict that increased microenvironmental complexity requires greater sperm density to maintain effective diffusive search and timely egg contact” - as mentioned in the above several points - these are not the points that need any numerical simulations to make predictions. At best those are numerical illustrations, and maybe the way to get a feeling of the quantitative effect.

Response: We agree. But we also feel that numerical illustrations may aid interpretation by more empirically minded biologist readers.

27. My final point is that in the extensive discussion section there are no qualitative explanations of the model results, which, in fact, can be argues in terms of the random walk theory. In terms of numbers (times/scales) there is no discussion of relevance to those to the underlying biological problem. Furthermore, the connection of the model results to the problem of selection and pressure need a dedicated explanation, why the quantifiers/measure are the one to use? What are the alternatives, what are the advantages/disadvantages?

Response: We have revised the discussion to place the existing literature on random walks in context, as well as expanded our discussion of the implications for these measures. As mentioned earlier, we haven’t expanded in detail on the more nuanced relationships between the selection aspects of the simulations and the analytical properties of random walks, because we are still working those details out and will address them in an upcoming separate manuscript.

28. So taken together, as you see, there is an extensive list of criticism on the technical implementation and its interpretation which need to be resolved before reconsidering this manuscript for publication.

Response: We very much appreciate the thorough review and feedback. We hope that we have addressed each concern appropriately, but are more than happy to revise further if there are any additional concerns.
